# Improving NonViral Gene Delivery Using MHz Bursts of Nanosecond Pulses and Gold Nanoparticles for Electric Field Amplification

**DOI:** 10.3390/pharmaceutics15041178

**Published:** 2023-04-07

**Authors:** Eivina Radzevičiūtė-Valčiukė, Jovita Gečaitė, Augustinas Želvys, Auksė Zinkevičienė, Rokas Žalnėravičius, Veronika Malyško-Ptašinskė, Aušra Nemeikaitė-Čenienė, Vytautas Kašėta, Natalija German, Jurij Novickij, Almira Ramanavičienė, Julita Kulbacka, Vitalij Novickij

**Affiliations:** 1 Department of Immunology, State Research Institute Centre for Innovative Medicine, 08406 Vilnius, Lithuania; eivina.radzeviciute@imcentras.lt (E.R.-V.); jovita.gecaite@stud.vu.lt (J.G.); augustinas.zelvys@imcentras.lt (A.Ž.); aukse.zinkeviciene@imcentras.lt (A.Z.);; 2 Faculty of Electronics, Vilnius Gediminas Technical University, 10223 Vilnius, Lithuania; veronika.malysko-ptasinske@vilniustech.lt (V.M.-P.);; 3 State Research Institute Center for Physical Science and Technology, 02300 Vilnius, Lithuania; rokas.zalneravicius@ftmc.lt; 4 Department of Biomodels, State Research Institute Centre for Innovative Medicine, 08406 Vilnius, Lithuania; 5 Department of Molecular and Cellular Biology, Faculty of Pharmacy, Wroclaw Medical University, 50-367 Wroclaw, Poland

**Keywords:** plasmid DNA, transfection, electroporation, GFP, CHO-K1

## Abstract

Gene delivery by the pulsed electric field is a promising alternative technology for nonviral transfection; however, the application of short pulses (i.e., nanosecond) is extremely limited. In this work, we aimed to show the capability to improve gene delivery using MHz frequency bursts of nanosecond pulses and characterize the potential use of gold nanoparticles (AuNPs: 9, 13, 14, and 22 nm) in this context. We have used bursts of MHz pulses 3/5/7 kV/cm × 300 ns × 100 and compared the efficacy of the parametric protocols to conventional microsecond protocols (100 µs × 8, 1 Hz) separately and in combination with nanoparticles. Furthermore, the effects of pulses and AuNPs on the generation of reactive oxygen species (ROS) were analyzed. It was shown that gene delivery using microsecond protocols could be significantly improved with AuNPs; however, the efficacy is strongly dependent on the surface charge of AuNPs and their size. The capability of local field amplification using AuNPs was also confirmed by finite element method simulation. Finally, it was shown that AuNPs are not effective with nanosecond protocols. However, MHz protocols are still competitive in the context of gene delivery, resulting in low ROS generation, preserved viability, and easier procedure to trigger comparable efficacy.

## 1. Introduction

Gene therapy or gene vaccination is a promising approach for the treatment of cancer, which shows good efficacy both in vivo and in applied clinical research [1,2,3]. Currently, the application of viruses is an effective method for DNA transfection, but it is associated with increased cytotoxicity, delivered gene size limitations, immunogenicity, or tumorigenesis [4,5,6]. Nonviral gene transfection methods have several advantages over viral methods, including lower immunogenicity, reduced risk of insertional mutagenesis, and better flexibility in terms of the size and type of genetic material that can be delivered. In recent years, there have been several new developments in the nonviral gene transfection context, including lipid-based nanoparticles [7], polymer-based delivery systems [8], CRISPR-Cas9 delivery [9], ultrasound [10], magnetofection [11], and electroporation [12], where electroporation is one of the most established ones [13,14]. Electroporation enhances reporter plasmid delivery to the skin to a greater extent than liposome conjugation, which makes it a safe and reliable method for obtaining efficient and effective delivery of plasmid DNA [15].

Electroporation is a pulsed electric field-mediated phenomenon that is based on cell plasma membrane polarization [16,17]. Due to polarization, a critical transmembrane voltage (TMP) is induced, and the hydrophilic pores are formed in the cell membrane facilitating drug and gene delivery [18]. The process can be reversible (i.e., the pores reseal over time) or irreversible when the cells die due to the severity of the membrane damage caused by pulsed electric fields (PEF) [19]. As a result, modulation of pulsed electric field parameters and optimization of the protocols for drug and gene delivery is a topic that does not lose actuality [20], ensuring good application capabilities in clinics [21,22]. Currently, the field of reversible electroporation (incl. electrotransfection) is dominated by the microsecond and millisecond range of pulses [13,23]. The European Standard Operating Procedures for Electrochemotherapy and Electrogenetherapy (ESOPE) involve sequences of 8 × 100 µs pulses delivered at 1 Hz [24]. Nevertheless, in the context of gene delivery, the electrophoretic component was considered to be crucial [25,26]; thus, the high voltage (HV) pulses are frequently supplemented with low voltage (LV) millisecond pulses to improve the electrotransfection efficacy [27,28]. Alternatively, the whole burst of pulses consists of 1–10 ms waveforms [29]. The application of long pulses (µs-ms) ensures efficient electrogene transfer; however, it comes with an array of associated negative effects. Longer pulses are responsible for the generation of reactive oxygen species (ROS) due to electrolysis [30,31] and increased muscle contractions (and potentially pain) [32,33]. Moreover, (if not accounted for) the thermal influence (i.e., Joule heating) might affect the treatment outcome [34]. Therefore, methods to improve electrotransfection efficacy without the requirement to increase the pulse duration are constantly researched.

The application of nanotechnology and nanoparticles in the area of drug and gene delivery is heavily exploited. For example, functionalized metal nanoparticles improve cytotoxicity in the context of cancer [35] or can be used to overcome the chemoresistance in cancer cells [36]. It is also agreed that a combination of nanoparticle-based gene therapy with a drug, radiotherapy, photodynamic therapy, immunotherapy, or others will be emphasized in future clinical studies [37]. In the context of electroporation, the theoretical model predicting the potential of the use of conductive nanoparticles emerged and was proposed by Lekner [38] and Qiu et al. [39]. It was shown that the conductive nanoparticles in close proximity to the membrane are capable of increasing the electric field and increasing the density of the hydrophilic pores. Applied in vitro research supported the in silico data [40,41,42,43]. Currently, there are less than 10 papers focusing on the phenomenon, and the array of parametric protocols is still limited to µs-ms protocols. Application of shorter pulses (i.e., nanosecond range) is extremely limited in the area of electrogene transfer, presumably due to the lack of significant electrophoretic components and the complexity of pulse generators. Nevertheless, the proof of concept was shown recently [13,44], and as expected, ultrashort pulses are less efficient for gene delivery, however, only when delivered with several Hz or kHz frequencies. In our previous studies, we have shown that compressing nanosecond pulses into an MHz burst triggers a new phenomenon of residual TMP accumulation, which significantly improves electrotransfer [45,46] and, thus, can be applied in nanosecond range electrogene delivery context [14].

In this work, we aimed to show the capability to improve gene delivery using MHz bursts of nanosecond pulses and characterize the potential use of gold nanoparticles (AuNPs) in this context. Additionally, we compared the generation of ROS accompanying the treatment and the effects of AuNPs on the process using both the standard (ESOPE-like) protocols, nanosecond protocols (kHz repetition frequency), and the new modality of nanosecond pulses (delivered at MHz frequency). The results of our work improve the understanding of polarization-based phenomena in the nanosecond range and have direct applicability for the development of new clinical gene transfer protocols based on nano-electroporation, which was considered not possible several years ago.

## 2. Materials and Methods

### 2.1. Electroporation Setup and Parameters

A 3 kV, 100 ns–1 ms square-wave pulse generator (VilniusTECH, Vilnius, Lithuania) [47] and a commercially available electroporation cuvette with a 1 mm gap between electrodes (Biorad, Hercules, CA, USA) were used in the experiments. The voltage applied to the cuvette was from 0.06 to 0.7 kV, corresponding to a 0.6–7 kV/cm electric field. The treatment included ESOPE-like µsPEF protocols (100 µs × 8, 1 Hz) with 0.6–1.4 kV/cm PEF amplitude. The nsPEF protocols were based on bursts of 100 pulses delivered at 1 kHz or 1 MHz frequency (3/5/7 kV/cm × 300 ns × 100).

### 2.2. Cell Culture

Chinese hamster ovary (CHO-K1) cells were cultivated in Petri dishes with RPMI 1640 medium enriched with 10% fetal bovine serum (FBS), 100 U/mL penicillin, 100 mg/mL streptomycin, and glutamine. The cell line CHO-K1 (ATCC CCL-61) was acquired from the national collection of the State Research Institute for Innovative Medicine (Vilnius, Lithuania). All cell culture reagents were derived from Gibco, Thermo Fisher Scientific, Waltham, MA, USA. Conditions for cell growth were 5% CO_2_, 37 °C.

On an experimental day, the cells were trypsinized (Thermo Fisher Scientific, Waltham, MA, USA), centrifuged, and resuspended in the electroporation buffer (sucrose 242 mM, Na_2_HPO_4_ 5.5 mM, NaH_2_PO_4_ 3 mM, MgCl_2_ 1.7 mM, pH 7.1) at concentration 6 × 10^6^ cells/mL. For the cell permeabilization and viability assays, cells were resuspended in the electroporation buffer at a concentration of 2 × 10^6^ cells/mL.

### 2.3. Cell Permeabilization

**Figure 1:**
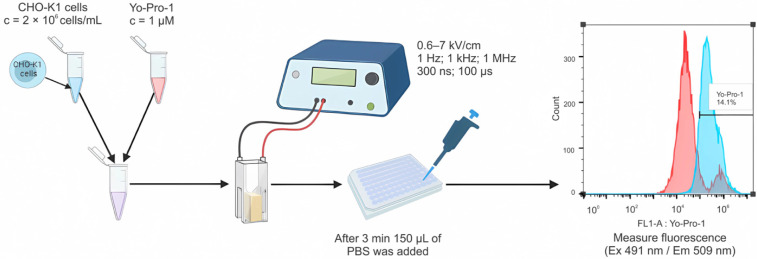
A scheme of cell membrane permeabilization experiments including gating strategy and permeabilized cells analysis. Blue color spectra indicate a shift in flureoscence intensity (Yo-Pro-1 positive cells); red—non-electroporated cells.

To test the efficiency of cell permeabilization, the green fluorescent stain Yo-Pro1 (Sigma-Aldrich, St. Louis, MO, USA) was used. Cells in electroporation buffer at a concentration of 2 × 10^6^ cells/mL were combined (or not) with AuNPs of various sizes (25 µg/mL) and mixed with Yo-Pro1 to a concentration of 1 μM. Samples were placed between the electrodes, and different protocols of PEF were used. After 3 min incubation at room temperature, 150 μL of phosphate-buffered saline (PBS) was added. Samples were analyzed using BD Accuri C6 flow cytometer (BD Biosciences, San Jose, CA, USA), where Yo-Pro1 (491⁄509) fluorescence was detected in channel FL1 (533/30 nm BPF). Results were analyzed with FlowJo software (BD, Franklin Lakes, NJ, USA). The scheme of cell permeabilization assay and analysis is shown in Figure 1.

The experiments were performed with and without AuNPs.

### 2.4. Cell Electrotransfection

**Figure 2:**
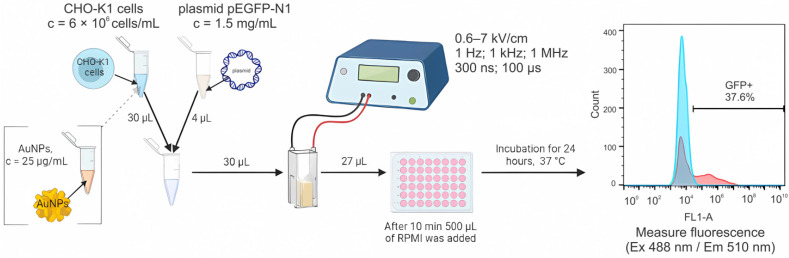
A scheme of electrotranfection experiments including gating strategy. Red color spectra indicate a shift in flureoscence intensity (GFP positive transfected cells); blue—non-electroporated cells.

The efficacy of electrotransfection was evaluated using pEGFP-N1 plasmid (4.7 kbp) encoding the green fluorescent protein (GFP). For each sample, 30 μL of cells were mixed with 4 μL of the plasmid DNA (purified with HiPure Expi Plasmid Gigaprep Kit (Thermo Fisher Scientific, Waltham, MA, USA)). All the steps were performed on ice. Different PEF protocols with and without AuNPs were used. After electroporation, the samples were transferred to a 48-well plate, and after 10 min of incubation, 500 μL of RPMI medium was added. The experimental plate was placed in an incubator for 24 h at 37 °C, 5% CO_2_. After incubation, the cells were trypsinized, collected in microtubes, centrifuged, and suspended in 90 μL PBS. The samples were transferred to a 96-well round-bottomed plate to be measured with a BD Accuri C6 and Amnis FlowSight (Luminex, Northbrook, IL, USA) flow cytometers. The fluorescence of transfected cells was detected using channel FL1 (533/30 nm BPF) and channel 2 (532/55 nm), respectively. The scheme of electrotransfection analysis is illustrated in Figure 2.

### 2.5. Viability Assay

Electrotransfection was performed as described above. After electroporation, samples were transferred into 96-well flat-bottomed plates and incubated for 10 min on ice, followed by the addition of growth media to the final 200 μL volume in each well. After 24 h, wells were washed twice with PBS, and to each well, 90 µL of PBS and 10 µL of cell viability reagent (PrestoBlue, Thermo Fisher Scientific, Waltham, MA, USA) were added. The cells were kept in an incubator for 2 h, and the fluorescence was measured using a Synergy 2 microplate reader and the Gen5 1.04.5 software (BioTek, Winooski, VT, USA). The excitation wavelength was 540/20 nm, and the emission was evaluated at 620/40 nm.

### 2.6. Fluorescence Microscopy

Immediately after electrotrasfection, a sample of CHO-K1 cells was seeded and grown on a sterile glass coverslip in 6-well plates (TPP, Switzerland) at a concentration of 2 × 10^5^ cells/mL in RPMI 1640 medium with 10% FBS, 100 U/mL penicillin, 100 μg/mL streptomycin, and 2mM L-glutamine (Gibco, Thermo Fisher Scientific, Waltham, MA, USA). Then cells were incubated for 24 h at 37 °C, 5% CO_2_. After incubation, glass coverslips were gently washed with PBS ×1 (Gibco, Thermo Fisher Scientific, Waltham, MA, USA) and placed on a microscope slide. Afterward, the cells were imaged using an Olympus IX81 inverted fluorescence microscope (Olympus, Hamburg, Germany), ORCA-ER digital camera (Hamamatsu, Japan), and Cell^M microscope system (Olympus, Hamburg, Germany).

### 2.7. Synthesis and Characterization of Gold Nanoparticles

Materials: All reagents utilized were of analytical grade and used as received without additional purification. Hydrogen tetrachloroaurate(III) trihydrate (HAuCl_4_·3H_2_O) and tannic acid were obtained from Carl Roth GmbH&Co (Karlsruhe, Germany), trisodium citrate—from Penta (Praha, Czech Republic). Deionized (DI) water (18 MΩ·cm) purified by Merck Millipore (Billerica, MA, USA) was used for all synthesis of different-sized AuNPs. 

AuNPs of 13 nm size were synthesized by reducing HAuCl_4_ by trisodium citrate in the presence of tannic acid according to the modified Turkevich method [48] and earlier described methodology [49]. Briefly, 80 mL of 0.0125% [*w*/*v*] of HAuCl_4_ solution and a mixture of 20 mL consisting of 4 mL 1% [*w*/*v*] trisodium citrate and 0.025 mL of 1% [*w*/*v*] tannic acid solutions in the deionized water were prepared and heated up to 60 °C in the separate Erlenmeyer flasks on a magnetic stirrer with electrical heating. Then these solutions were mixed, heated up to 98 °C under stirring (1000 rpm), and kept at this temperature for 3 min to yield a red color solution of AuNPs. The colloid solution of AuNPs was stored in the dark at +4 °C. The size of synthetized AuNPs (dry sample) determined by atomic force microscopy using tapping mode was within the range of 12–16 nm [50]. The characterization of AuNPs by dynamic light scattering technique showed that AuNPs are nearly monodispersed since the determined diameter was 12.96 ± 0.607 nm. Zeta potential measurements reveal that 13 nm AuNPs have an average surface charge of −34.2 ± 2.3 mV in the obtained colloidal solution. The initial concentration of gold (according to mass) used for the synthesis of 13 nm AuNPs was 50 µg/mL.

**Figure 3:**
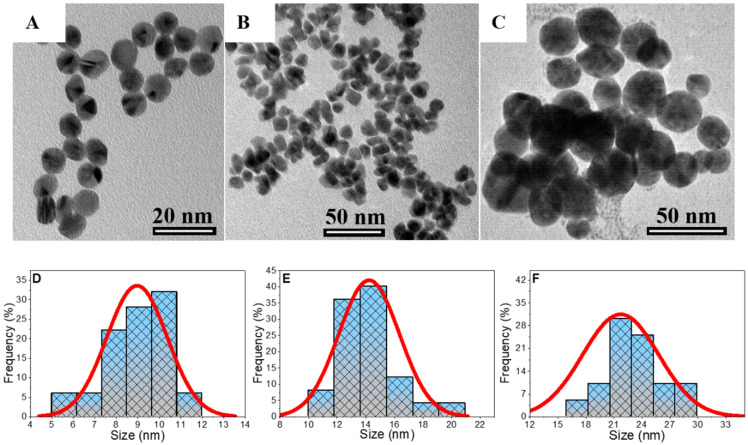
TEM images and size distribution histograms of small (**A**,**D**), medium (**B**,**E**), and large (**C**,**F**) AuNPs synthesized via citrate-induced reduction in tetrachloroauric acid at 70, 80, and 90 °C, respectively. The size distribution of AuNPs was inspected from TEM images by counting at least 100 accidentally selected AuNPs.

In order to compare the effects of AuNPs size on the electroporation and electrotransfection efficiency, new stocks of colloidal solutions were prepared using the Turkevich method. To maintain the set temperatures, AuNPs were synthesized in a thermostat-controlled 100 mL glass reaction vessel wrapped in aluminum foil. Briefly, the reaction vessel was filled with 50 mL of 1 mM HAuCl_4_·solution and kept to yield (approximately 20 min) the temperature values of 70, 80, and 90 °C, respectively. Then, 10 mL of a freshly prepared and warmed solution of 38.8 mM trisodium citrate was injected into the reaction vessel under vigorous stirring and kept for 10, 15, and 30 min. The small AuNPs with a diameter of ~6–9 nm was synthesized at 70 °C for 10 min, whereas the medium-sized (10–15 nm) and higher (17–25 nm) nanoparticles were grown by keeping the higher temperatures at 80–90 °C and more extended reaction times of 15–30 min, respectively. Finally, the reaction vessel was transferred into the ice bath to stop the reduction reaction and kept there until the red-wine-colored solution decreased to room temperature. The obtained red-wine-colored solutions were placed into the fridge if not in use to prevent possible contamination. The morphology (Figure 3A–C) and size distribution (Figure 3D–F) of the as-synthesized different-sized AuNPs were investigated by transmission electron microscope (TEM) FEI Tecnai F20 X-TWIN (Eindhoven, The Netherlands) equipped with a field emission gun. The colloidal solutions of AuNPs were dispersed in ethanol, drop-casted onto a carbon-coated nickel grid, and left to dry under environmental conditions. TEM images were recorded using a Gatan Orius CCD camera (Pleasanton, CA, USA) in bright field mode at an acceleration voltage of 200 kV. The averaged particle diameter was estimated from TEM images by using ImageJ 1.51k software (National Institutes of Health, WI, USA). The size of AuNPs was also assessed by measuring the surface plasmon resonance of colloidal solutions using Lamda 35 UV/VIS spectrophotometer (Perkin Elmer, Waltham, MA, USA). The concentration of gold in the colloidal solutions was determined by inductively coupled plasma optical emission spectrometer (ICP-OES) OPTIMA 7000DV (Perkin Elmer, Waltham, MA, USA). Before the measurements, AuNPs were centrifuged and resuspended in DI water to remove the gold acid residues. The AuNPs were dissolved in aqua regia solution and further subjected to ICP-OES measurements performed on emission peaks at λ_Au_ = 267.595 nm.

The size distribution histograms of AuNPs enable to estimate size intervals of three types of nanoparticles that are further assigned as small (6–12 nm), medium (10–20 nm), and large (14–29 nm) with averaged particles radius of 9, 14, and 22 nm, respectively (Figure 3D–F).

Since the noble metal-based NPs (i.e., gold and silver) exhibit strong surface plasmon resonance (SPR) and show size-dependent characteristics [51], the red-wine-colored solutions of different-sized AuNPs were subjected to UV-VIS analysis to gain more information about colloidal nanoparticles size. The particle radius was estimated by following the Haiss et al. investigations [52]. According to the (1) equation, the particle size (nm) was calculated by measuring the ratio of the absorbance at the ratio of SPR peak (A_spr_) to the registered averaged absorbance at 450 nm wavelength (A_450_), whereas B1 and B2 are experimentally estimated parameters (3.0 and 2.2, respectively).

(1) $$d=\exp\left(B_{1}\frac{A_{spr}}{A_{450}}-B_{2}\right)$$

**Figure 4:**
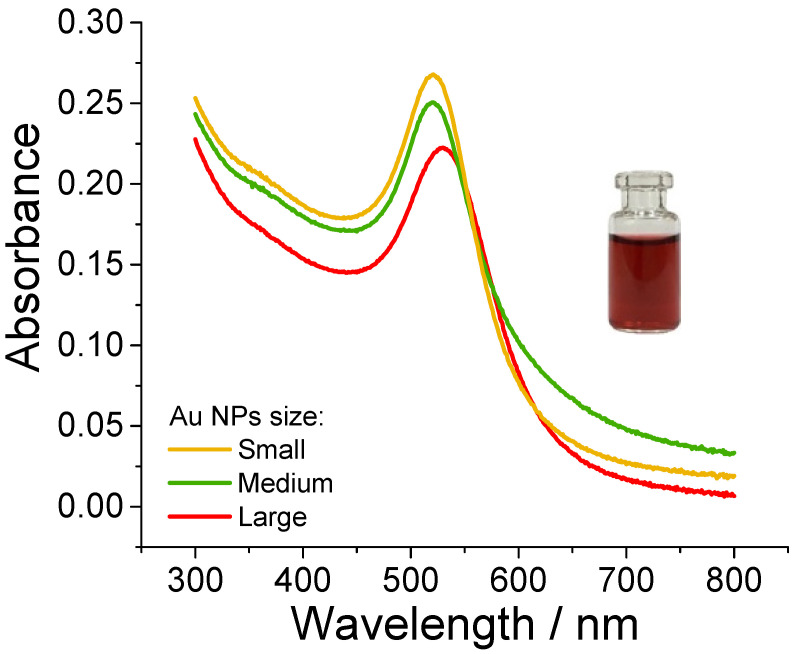
Absorbance spectra of small (Ø_avg_~9 nm), medium (Ø_avg_~14 nm), and large (Ø_avg_~22 nm) AuNPs. Insets: as-synthesized red-wine colored AuNPs colloidal solution.

Figure 4 displays the absorbance spectra of different-sized AuNP colloidal solution with the A_spr_ peaks at 521, 522, and 530 nm for small, medium, and large nanoparticles.

The calculated particles size with the radius of 2, 10, and 20 nm from UV-VIS are slightly smaller than those estimated by TEM. Based on our previous research [53] to prepare AuNPs using the identical protocol, the citrate-capped AuNPs have a negative surface charge ranging from −39 to −44 mV, respectively. For all the AuNPs used in the study, the aliquot of colloidal solutions was further diluted with citrate buffer to obtain identical content of AuNPs (50 μg/mL) and was used for further research by diluting the stock 1:1 with cell suspension (final concentration of 25 μg/mL).

The characteristics of AuNPs used in the study are provided in Table 1.

### 2.8. Modelling of Electric Field and Nanoparticles Using FEM

**Figure 5:**
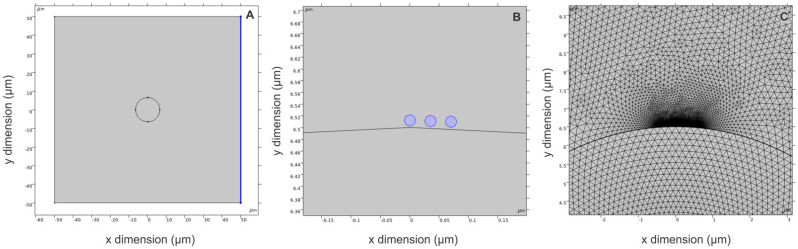
FEM model in COMSOL environment, where (**A**) cell model inside a cuvette; (**B**) model of gold nanoparticles in close proximity with cell membrane; (**C**) generated mesh structure.

A cell polarization model based on the finite element method (FEM) was developed in COMSOL (COMSOL Multiphysics, Sweden) using 2D geometry. The cell was modeled as a conductive sphere (σ_c_ = 0.3 S/m [54], radius of 6.5 μm) with a thin layer (5 nm) of low electrical conductivity (1.1 × 10^−7^ S/m [55]) representing a lipid membrane similar to the previous study [56]. Briefly, the extracellular medium (0.1 S/m) was approximated as a cube with a 1 mm edge, and the cathode/anode was defined as aluminum electrodes (1 mm gap). A free triangular mesh with 6.9M domain elements and 2975 boundary elements was generated. The 120 V voltage (100 µs) was defined as an input (corresponding to 1.2 kV/cm PEF). The nanoparticles were programmed as gold spheres (using the COMSOL library of materials) with a size of 20 nm. The mesh structure and the cell model are shown in Figure 5.

The minimum and maximum element sizes were selected as 1 and 200 nm, respectively. A maximum element growth rate of 1.1 was defined. The effects of AuNPs on the spatial electric field distribution were studied.

### 2.9. ROS Analysis

For ROS evaluation, the cells in an electroporation medium were incubated with H_2_DCFDA (DCF) (Sigma-Aldrich, Chemie GmbH, Steinheim, Germany) dye at a concentration of 10 μM for 30 min. Afterward, 50 μL (4 × 10^6^ cells/mL) of cell/dye suspension with or without AuNPs was placed into the electroporation cuvette and treated by PEF. After the treatment, the samples were transferred to a black 96-well plate (Thermo Fisher Scientific, Waltham, MA, USA) and incubated for an additional 20 min at room temperature. Then 100 μL of 97% ethanol was added to each sample, and the samples were incubated for another 10 min. Then DCF fluorescence measurements were performed using Synergy 2 microplate reader and Gen5 1.04.5 software (BioTek, Winooski, VT, USA).

### 2.10. Statistical Analysis

One-way analysis of variance (ANOVA; *p* < 0.05) was used to compare different treatments. Tukey HSD multiple comparison test for the evaluation of the difference was used when ANOVA indicated a statistically significant result (*p* < 0.05 was considered statistically significant). Each experimental point was obtained from at least three independent experiments, and results are represented as mean ± standard deviation.

## 3. Results

### 3.1. PEF Induced Electropermeabilization

**Figure 6:**
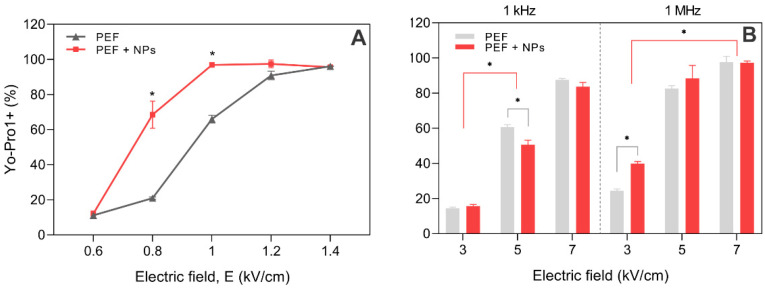
Permeabilization of CHO-K1 cells with the pulsed electric field, where (**A**) ESOPE protocols (100 μs × 8); (**B**) nanosecond burst protocols (300 ns × 100). PEF—pulsed electric field; NPs—gold nanoparticles (13 nm). Asterisk (*) corresponds to statistically significant differences (*p* < 0.05).

Firstly, in order to characterize electroporation with and without AuNPs (13 nm), the permeabilization of the cell membrane was evaluated using a YO-PRO-1 fluorescent marker, and the results are presented in Figure 6. The ESOPE protocols (100 μs × 8) without AuNPs have a scaling in permeabilization efficiency, which is dependent on the pulse amplitude. The introduction of AuNPs significantly improves the number of permeabilized cells at the same pulsing conditions.

Equivalent pulsing conditions can be derived for nanosecond range protocols (Figure 6B). We observed that a 7 kV/cm amplitude is required to trigger more than 80% permeabilization, while for nanosecond compressed pulse (1 MHz) bursts, the same rate of permeabilization can be triggered already at 5 kV/cm. Interestingly, the effect of AuNPs on nanosecond electropermeabilization is less profound when compared to ESOPE, and in most cases, the difference is not statistically significant. In order to ensure high electrotransfection efficiency, a high permeabilization rate must be triggered; thus, the protocols triggering more than 80% permeabilization are of utmost interest.

### 3.2. Electrotransfection of CHO-K1 Cell Line

**Figure 7:**
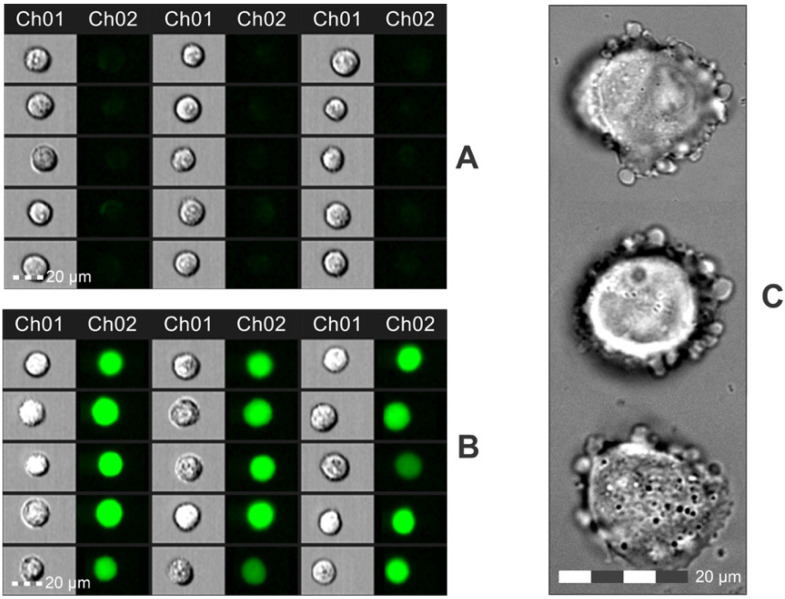
Photographs of cells 24 h post-electrotransfection, where (**A**) PEF nontreated control (plasmid only), 15 accidentally selected cells; (**B**) cells treated with 7 kV/cm × 300 ns × 100 pulses (1 MHz), 15 accidentally selected cells; (**C**) images of cell swelling and blebbing after PEF treatment captured using 60 × UPlanSApo oil objective (NA = 1.35) on an Olympus IX81 microscope (Olympus, Hamburg, Germany). Ch01—brightfield images and Ch02—fluorescence images using a laser of 488 and 505–560 nm BP filter.

The electrotransfection efficiency was evaluated 24-h post-treatment, and firstly, the cells were analyzed in terms of morphology changes using Amnis FlowSight and microscopy. The photographs of cells are shown in Figure 7.

**Figure 8:**
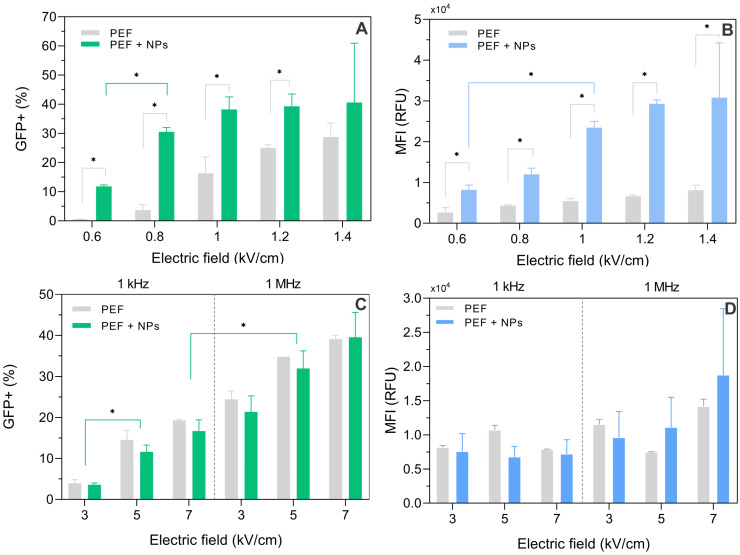
Electrotransfection of CHO-K1 with and without AuNPs, where (**A**) electrotransfection efficiency using ESOPE protocols; (**B**) median fluorescence intensity using ESOPE electrotransfection; (**C**) nsPEF induced electrotransfection; (**D**) median fluorescence intensity induced by nsPEF. Asterisk (*) corresponds to statistically significant difference (*p* < 0.05).

As reported in Figure 7A,B, the cells feature a high fluorescence signal indicating successful electrotransfection. It should be noted that in all of the electroporated samples, there were some cells (<5%) featuring swelling and blebbing (Figure 7C), which was not the case in the untreated control samples. The cytotoxicity of electric fields is a known issue; therefore, the pulses should be adjusted, respectively, to cause predominantly reversible electroporation. For quantitative analysis, both the number of fluorescent cells and the median fluorescence intensity (MFI) were analyzed further in the study, and the results are summarized in Figure 8.

We observed that with ESOPE protocols (Figure 8), the electrotransfection rate steadily increased with the increase in pulse amplitude (up to 30+%). However, when AuNPs are added, the 1–1.4 kV/cm range trigger a saturated electrotransfection efficiency (i.e., an increase in pulse amplitude does not result in an increase in electrotransfection rate), which is in agreement with the permeabilization data (Figure 6A). It is shown that the application of AuNPs can significantly improve gene delivery. The median fluorescence of transfected cells is also significantly increased when AuNPs are added (Figure 8B), which indicates that the number of DNA complexes is higher. Nevertheless, it is not the case for nanosecond protocols (Figure 8C,D). The AuNPs have no tendency to increase the electrotransfection efficiency in the nanosecond pulse range, which is also in agreement with the permeabilization data (Figure 6B). The median fluorescence after nanoprotocols is, on average, higher than ESOPE alone for most of the protocols employed in the study but several-fold lower if ESOPE is combined with AuNPs.

### 3.3. Viability Results

**Figure 9:**
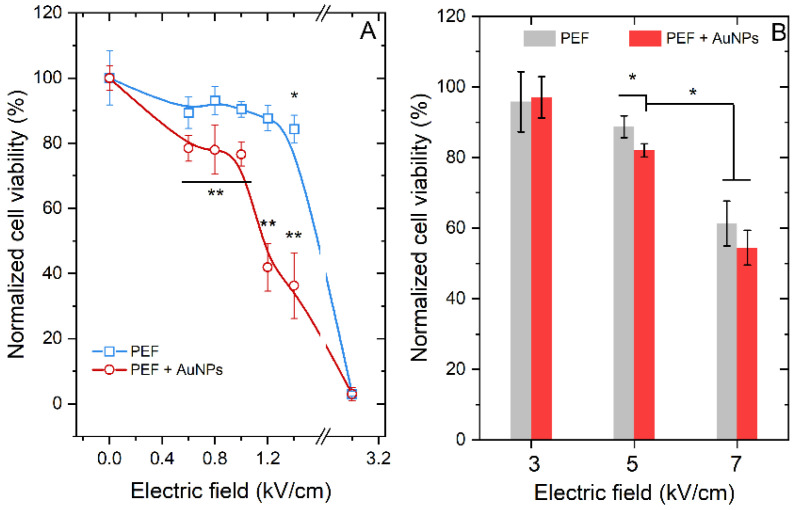
Viability of cells following electrotransfection with and without AuNPs, where (**A**) ESOPE protocols; (**B**) MHz burst of nanosecond pulses. Asterisk (*) corresponds to statistically significant difference (*p* < 0.05) versus untreated control, unless marked differently. Asterisk (**) corresponds to statistically significant difference (*p* < 0.05) versus PEF only treatment.

The preserved viability of cells is of utmost importance in electrotransfection experiments; therefore, the viability of the cells was also evaluated. The results are summarized in Figure 9.

Due to the field amplification, the ESOPE protocols are more cytotoxic with AuNPs than without; the 1.2 and 1.4 kV/cm electric field protocols result in more than 60% loss of cell viability (Figure 9A). The 1 kV/cm occurs to be optimal due to saturated electrotransfection (refer to Figure 8A) and minimal losses (<20%) in cell viability. As expected, the AuNPs have no effect on the cell viability for nanosecond range protocols, which is in agreement with the electropermeabilization and electrotransfection data. The optimal protocol is the 5 kV/cm × 300 ns × 100 pulses delivered at 1 MHz frequency.

### 3.4. Evaluation of ROS

**Figure 10:**
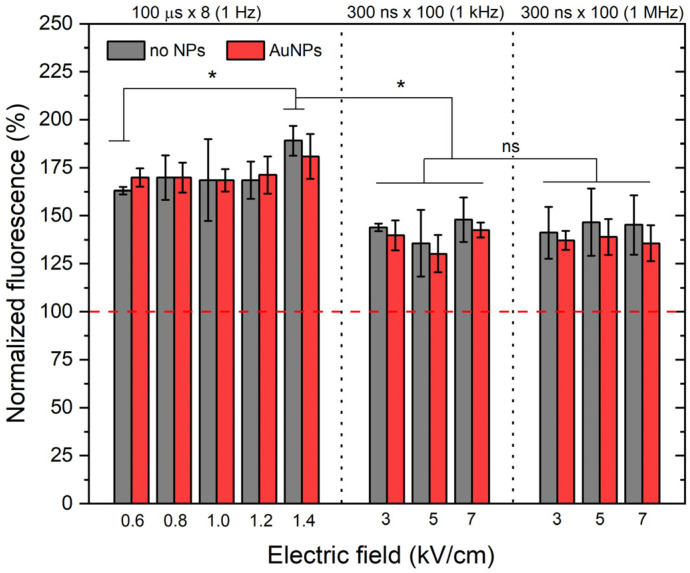
Generation of ROS during electroporation-based treatment. Asterisk (*) corresponds to statistically significant difference (*p* < 0.05; ns—non-significant).

Further, the ROS generation due to PEF treatment was evaluated, and the results are summarized in Figure 10. The long microsecond pulses result in a more than 60% increase in DCF fluorescence, indicating higher ROS and, thus, potentially higher oxidative damage. The 1.4 kV/cm protocols resulted in a more than 80% increase when compared to the control, which is an expected result since the electric current is higher, resulting in higher electrolysis. The effects of AuNPs on the additional ROS generation during PEF treatment were not detected or lost within the standard deviation of data.

Nanosecond protocols resulted in a significantly lower ROS generation independently of the burst frequency. The effects of AuNPs on ROS generation during nano-electroporation were also not detected.

### 3.5. Dependence of the Effect on AuNP Size

**Figure 11:**
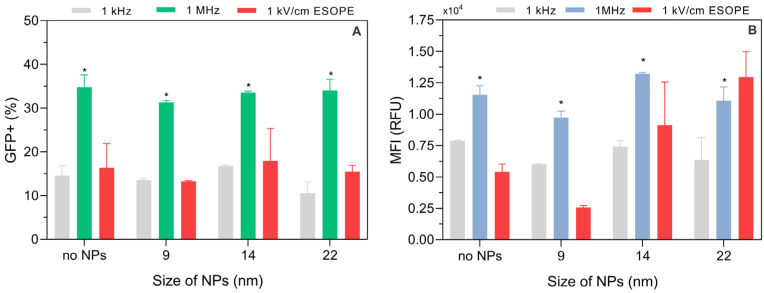
Dependence of electrotranfection efficiency on PEF parameters and AuNP size, where (**A**) percentage of GFP positive cells; (**B**) median fluorescence intensity. Asterisk (*) corresponds to statistically significant difference (*p* < 0.05).

The effects of AuNP size using different stocks of nanoparticles were studied. The experiments with 9, 14, and 22 nm AuNPs were performed using the most successful protocols determined in the study (i.e., 1 kV/cm × 100 μs × 8 and 5 kV/cm × 300 ns × 100). The results are summarized in Figure 11.

The effects of AuNPs during electroporation are dependent on the synthesis of the AuNPs. The stocks of 9, 14, and 22 nm resulted in a significantly weaker electrotransfection improvement (1 kV/cm ESOPE protocol) when compared to the 13 nm AuNP stock used in the study, which might be associated with the surface charge of the AuNPs. Nevertheless, a clear tendency in terms of median fluorescence intensity was observed (Figure 11B)—with the increase in the AuNP size, the MFI increases. The AuNPs, independently of the size, had no effect on electrotransfection when nanosecond pulses were employed. The 1 MHz pulses were the most efficient for nano-electrotransfection.

### 3.6. FEM Model Results

**Figure 12:**
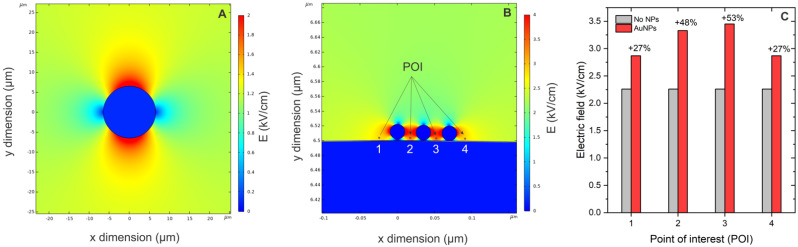
FEM modeling results, where (**A**) cell polarization model; (**B**) PEF amplification by AuNPs in close proximity with the membrane; (**C**) quantified expression of field amplification rate. POI corresponds to points of interest (1–4) where the field amplitude is evaluated.

Finally, to support the experimental data, the FEM simulation was performed, and the results are presented in Figure 12.

During PEF application, the cell membrane is polarized (Figure 12A); however, when the AuNPs are in close proximity to the membrane (Figure 12B), the amplitude is significantly amplified. The quantitative analysis is summarized in Figure 12C—more than 50% field amplification is expected, which is in agreement with the permeabilization and the electrotransfection data using ESOPE protocols.

## 4. Discussion

In this work, the effects of AuNPs on the efficacy of gene delivery using conventional electroporation protocols and nanosecond high-frequency bursts were studied. It was shown that AuNPs could amplify electric fields in close proximity to the cell membrane, which significantly improves the effectiveness of ESOPE protocols. It was not the case for nanosecond protocols, where all of the AuNPs employed in the study were ineffective.

It is established that the electrophoretic force has a positive effect on electrogene transfer [26]; thus, the area of electroporation-mediated electrotransfection is dominated by longer pulses or a combination of the short and long waveforms (i.e., HV/LV methodology). We have shown that the efficiency of gene delivery can be improved by AuNPs, which amplify the electric field according to the presented data and available knowledge. Electroporation is a threshold-type polarization phenomenon; therefore, an increase in the PEF amplitude results in an increase in pore size, density, and stability [39]. As a result, improvement of electrogene transfer in the ESOPE range is expected. In the context of nanosecond pulses, we speculate that the inefficacy of AuNPs is associated with the lack of an electrophoretic component of the nanosecond burst. Ghorbel et al. have shown [42] that the accumulation of conductive nanoparticles for the enhancement of cell electropermeabilization is associated with electrophoresis. Longer pulses increase the accumulation of either aggregated or individual NPs near the cell membrane, supporting the electrophoretic nature of the observed effects [42]. In the case of nanosecond pulses, the negatively charged NPs are electrostatically repelled from the cell membrane [57], and there is no electrophoretic component to compensate for this. This hypothesis explains the inefficiency of negatively charged AuNPs with nanosecond bursts and also partly explains why the AuNP stock with the average surface charge of −34.2 mV was significantly better when compared to the −39–44 mV surface-charged AuNPs. In order to compensate for this, positively charged conductive NPs should be used in the future to ensure repeatable close spatial proximity of NPs with the membrane via the electrostatic forces. Another solution is to use a low-voltage, but longer prepulse protocol followed by a high-frequency nanosecond burst. However, we believe that it is a suboptimal solution since it will increase ROS generation.

In terms of ROS, we have shown that the AuNPs have no effect on additional ROS generation during electroporation. At the same time, ESOPE protocols trigger ~20% higher ROS when compared to the nanosecond protocols used in the study. The result is in agreement with the available knowledge [31]. Minimizing ROS is a priority in the context of gene delivery due to the detrimental effects of oxidative damage on the plasmid [58].

In this study, we have also analyzed the effect of frequency during nanosecond bursts, and it was shown that 1 kHz pulses are inferior to 1 MHz protocols or ESOPE bursts. Compressing the pulses into an MHz burst triggers a phenomenon of residual TMP accumulation between the consequent pulses [45]. The capability to prevent complete depolarization of the cell membrane between the pulses positively affects the number and stability of the pores and, thus, improves electrotransfer. This phenomenon was recently confirmed in silico [59], in vitro [56,60], and in vivo [61]. Based on the results, we can conclude that the application of nanosecond pulses, which are compressed into an MHz burst, can be successfully used for gene delivery as a natural evolution of ESOPE procedures since the whole electroporation field is moving towards the shorter pulse duration range. The application of shorter pulses enables better control of input energy and minimizes muscle contractions, oxidative damage, and the negative effects of electric field inhomogeneity in the tissue.

We have also shown that the size of AuNPs has an effect on gene delivery during electroporation. Bigger nanoparticles (i.e., 22 nm) showed the highest increase in median GFP fluorescence intensity when compared to 14 or 9 nm AuNPs. The result partly supports the data by Miklavčič et al. [43].

Finally, it should be noted that AuNPs, while routinely used in biomedical applications, can cause skin pigmentation [62], which is undesired. Therefore, future studies with other noble metals, especially metal oxide nanoparticles (i.e., magnetic iron oxide NPs), which are cheap, have excellent biocompatibility, and can be synthesized with a positive surface charge, would be of great interest and deserve further development of electroporation-assisted gene delivery technology.

## 5. Conclusions

The gene delivery using ESOPE protocols can be significantly improved with AuNPs; however, the efficacy is strongly dependent on the surface charge of AuNPs and their size. The application of lower PEF amplitude during ESOPE minimizes ROS generation. At the same time, AuNPs are not effective with nanosecond protocols. However, MHz protocols are competitive in the context of gene delivery by themselves, resulting in low ROS generation, preserved viability, and easier procedure (do not require AuNPs) to trigger comparable efficacy.

## Figures and Tables

**Table 1:** Characteristics of AuNPs used in the study.

AuNP Size	ζ Potential	Final Experimental Concentration
13 nm	−34 mV	25 μg/mL
9 nm	from −39 to −44 mV	
14 nm		
22 nm		

## Data Availability

Data available from the corresponding author V.N. on request.
